# Supplementary material for: Fatty Pancreas Is a Risk Factor for Pancreatic Cancer: A Systematic Review and Meta-Analysis of 2956 Patients
Source: Cancers (Basel). 2023 Oct 7;15(19):4876. doi: 10.3390/cancers15194876 (PMC10571813; doi:10.3390/cancers15194876)
Supplement: Supplementary file 1 [file cancers-15-04876-s001.zip › cancers-2601989-supplementary.pdf]

Supplementary Materials: Fatty Pancreas is a Risk Factor for Pancreatic Cancer: A Systematic Review and Meta-Analysis of 2956 Patients

Mónika Lipp [1,2,3], Dorottya Tarján [1,2], Jimin Lee [2,4], Ádám Zolcsák [2,5], Eszter Szalai [2,6], Brigitta Teutsch [2,3], Nándor Faluhelyi [2,7], Bálint Erőss [1,2,3], Péter Hegyi [1,2,3,8,†] and Alexandra Mikó [2,3,9,*,†]

[1] Institute of Pancreatic Diseases, Semmelweis University, 1083 Budapest, Hungary; lipp.monika@semmelweis.hu (M.L.); tarjan.dorottya@semmelweis.hu (D.T.); eross.balint@semmelweis.hu (B.E.); hegyi.peter@semmelweis.hu (P.H.)
[2] Centre for Translational Medicine, Semmelweis University, 1085 Budapest, Hungary; ji.lee@stud.semmelweis.hu (J.L.); zolcsak.adam@med.semmelweis-univ.hu (Á.Z.); szalai.eszter@semmelweis.hu (E.S.); teutsch.brigitta@semmelweis.hu (B.T.); faluhelyi.nandor@pte.hu (N.F.)
[3] Institute for Translational Medicine, Medical School, University of Pécs, 7624 Pécs, Hungary
[4] Medical School, Semmelweis University, 1085 Budapest, Hungary
[5] Department of Biophysics and Radiation Biology, Semmelweis University, 1094 Budapest, Hungary
[6] Department of Restorative Dentistry and Endodontics, Semmelweis University, 1088 Budapest, Hungary
[7] Department of Medical Imaging, Medical School, University of Pécs, 7624 Pécs, Hungary
[8] Translational Pancreatology Research Group, Interdisciplinary Centre of Excellence for Research
Development and Innovation, University of Szeged, 6725 Szeged, Hungary
[9] Department of Medical Genetics, Medical School, University of Pécs, 7624 Pécs, Hungary
* Correspondence: miko.alexandra@pte.hu
† These authors contributed equally to this work.

Supplementary Materials
Documentum Legends:

Document S1. Search term

Document S2. Detailed effect measure and synthesis methods

Figure Legends:

Figure S1. Contour-enhanced funnel plot visualizing the odds ratio of fatty pancreas among pancreatic cancer patients and controls

Figure S2. Contour-enhanced funnel plot visualizing the proportion of fatty pancreas among pancreatic cancer patients

Table Legends:

Table S1. PRISMA checklist

Table S2. Risk of bias assessment –analysis A: risk of bias assessment on study level (a) and across studies (b)

Table S3. Risk of bias assessment – fatty pancreas among pancreatic cancer patients and controls: risk of bias assessment on study level (a) and across studies (b)

Table S4. Risk of bias assessment – pancreatic cancer population: risk of bias assessment on study level (a) and across studies (b)

Table S5. Evidence table of analysis A

Table S6. Evidence table of fatty pancreas among pancreatic cancer patients and controls

Table S7. Evidence table of pancreatic cancer population and control group

Document S1. Search term.

(pancreas OR pancrea*) AND (fatty OR fat OR steatosis OR lipoma* OR adipo*) AND (neoplas* OR cancer* OR malignan* OR tumor OR tumour OR carcin* OR adenocarcinoma) AND (EUS OR "endoscopic ultrasound" OR "endoscopic ultrasonography" OR CT OR "computed tomography" OR MRI OR MR OR "magnetic resonance" OR histology OR histological OR histopathologically OR patholo*)

Document S2. Detailed effect measure and synthesis methods.

A random intercept logistic regression model method - more specifically, a random intercept logistic regression model - was used to pool proportions (as recommended by Schwarzer et al. [1] and Stijnen et al. [2]). Pooled OR was calculated using the Mantel-Haenszel method [3,4]. The exact Mantel-Haenszel method (without continuity correction) was used to handle zero or "total" cell counts (as recommended [5,6]). We used a Hartung-Knapp adjustment ([7,8]) for CIs. This adjustment was applied only if it was more conservative than the classical one (as recommended by Jackson et al. [9] as a hybrid method 2). To estimate the measure of heterogeneity variance (τ^2), the maximum likelihood method of the prevalence measure was used. For OR calculation, the Paule-Mandel method [10] (recommended by Veroniki et al. [11]) was used with the Q profile method for the confidence interval. Prediction interval calculations were based on a t-distribution. For the forest plots, the Agresti-Coull method [12] was used to calculate the CI for the proportion of individual studies. For 0 or "total" cell counts, the individual study proportion and OR with 95% CI were calculated by adding 0.5 as continuity correction (used only for visualization in forest plot).

Figure S1. Contour-enhanced funnel plot visualizing the odds ratio of fatty pancreas among pancreatic cancer patients and controls.

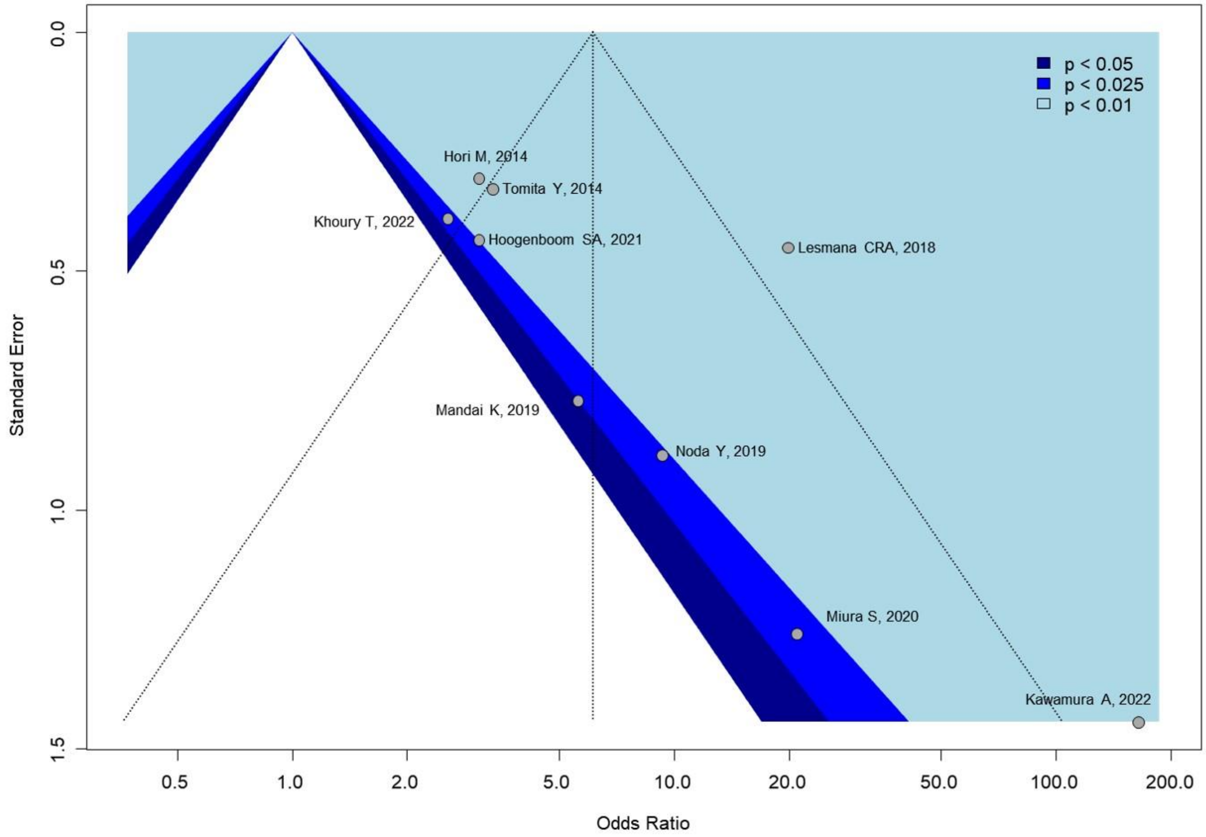

Figure S2. Contour-enhanced funnel plot visualizing the proportion of fatty pancreas among pancreatic cancer patients.

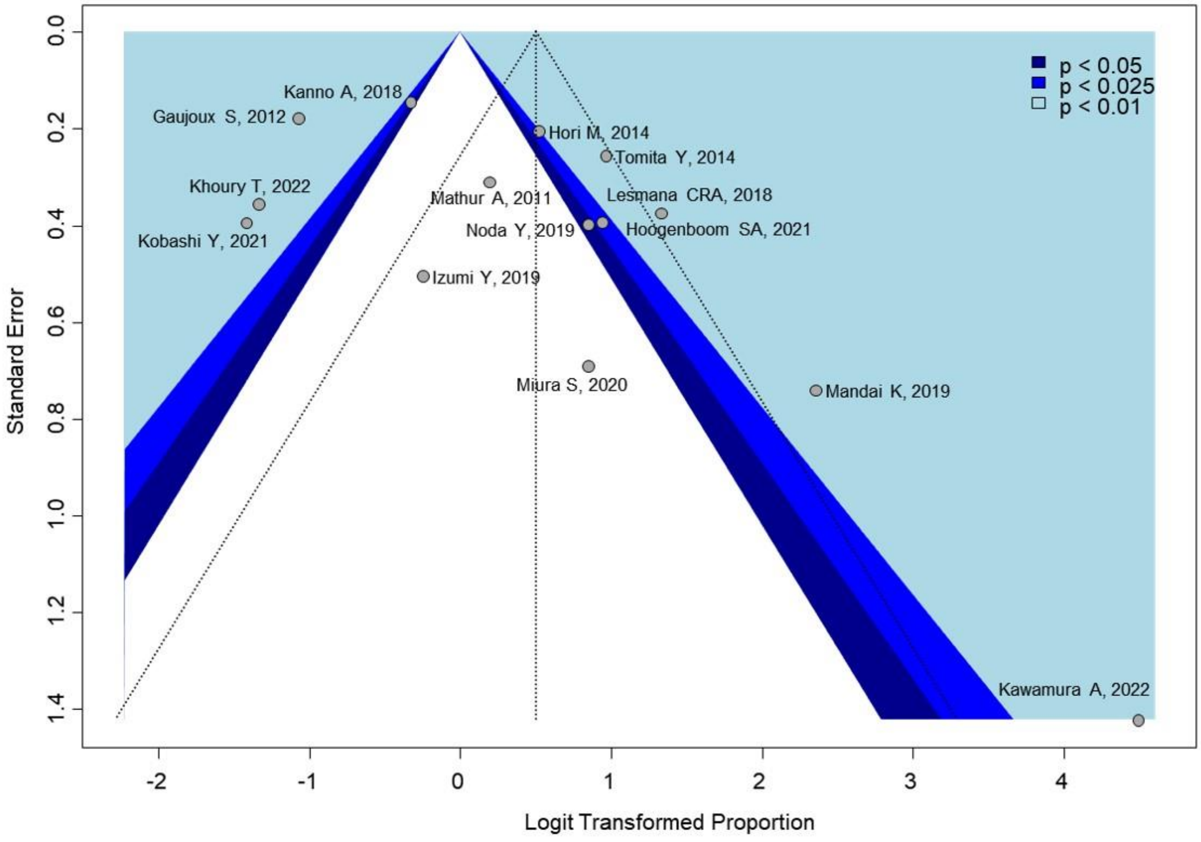

Table S1. PISMA 2020 checklist.

Section and Topic	Item #	Checklist item	Location where the item is reported
**TITLE**			
Title	1	Identify the report as a systematic review.	1
**ABSTRACT**			
Abstract	2	See the PRISMA 2020 for Abstracts checklist.	4
**INTRODUCTION**			
Rationale	3	Describe the rationale for the review in the context of existing knowledge.	6
Objectives	4	Provide an explicit statement of the objective(s) or question(s) the review addresses.	6
**METHODS**			
Eligibility criteria	5	Specify the inclusion and exclusion criteria for the review and how studies were grouped for the syntheses.	7
Information sources	6	Specify all databases, registers, websites, organisations, reference lists and other sources searched or consulted to identify studies. Specify the date when each source was last searched or consulted.	7
Search strategy	7	Present the full search strategies for all databases, registers and websites, including any filters and limits used.	7
Selection process	8	Specify the methods used to decide whether a study met the inclusion criteria of the review, including how many reviewers screened each record and each report retrieved, whether they worked independently, and if applicable, details of automation tools used in the process.	8
Data collection process	9	Specify the methods used to collect data from reports, including how many reviewers collected data from each report, whether they worked independently, any processes for obtaining or confirming data from study investigators, and if applicable, details of automation tools used in the process.	7
Data items	10a	List and define all outcomes for which data were sought. Specify whether all results that were compatible with each outcome domain in each study were sought (e.g. for all measures, time points, analyses), and if not, the methods used to decide which results to collect.	7
	10b	List and define all other variables for which data were sought (e.g. participant and intervention characteristics, funding sources). Describe any assumptions made about any missing or unclear information.	8
Study risk of bias assessment	11	Specify the methods used to assess risk of bias in the included studies, including details of the tool(s) used, how many reviewers assessed each study and whether they worked independently, and if applicable, details of automation tools used in the process.	8
Effect measures	12	Specify for each outcome the effect measure(s) (e.g. risk ratio, mean difference) used in the synthesis or presentation of results.	7
Synthesis methods	13a	Describe the processes used to decide which studies were eligible for each synthesis (e.g. tabulating the study intervention characteristics and comparing against the planned groups for each synthesis (item #5)).	8
	13b	Describe any methods required to prepare the data for presentation or synthesis, such as handling of missing summary statistics, or data conversions.	8
	13c	Describe any methods used to tabulate or visually display results of individual studies and syntheses.	8-9
	13d	Describe any methods used to synthesize results and provide a rationale for the choice(s). If meta-analysis was performed, describe the model(s), method(s) to identify the presence and extent of statistical heterogeneity, and software package(s) used.	8-9
	13e	Describe any methods used to explore possible causes of heterogeneity among study results (e.g. subgroup analysis, meta-regression).	8-9
	13f	Describe any sensitivity analyses conducted to assess robustness of the synthesized results.	9
Reporting bias assessment	14	Describe any methods used to assess risk of bias due to missing results in a synthesis (arising from reporting biases).	8
Certainty assessment	15	Describe any methods used to assess certainty (or confidence) in the body of evidence for an outcome.	9

Table S1. PISMA 2020 checklist.

Section and Topic	Item #	Checklist item	Location where the item is reported
**RESULTS**			
Study selection	16a	Describe the results of the search and selection process, from the number of records identified in the search to the number of studies included in the review, ideally using a flow diagram.	9
	16b	Cite studies that might appear to meet the inclusion criteria, but which were excluded, and explain why they were excluded.	9
Study characteristics	17	Cite each included study and present its characteristics.	9-10
Risk of bias in studies	18	Present assessments of risk of bias for each included study.	11
Results of individual studies	19	For all outcomes, present, for each study: (a) summary statistics for each group (where appropriate) and (b) an effect estimate and its precision (e.g. confidence/credible interval), ideally using structured tables or plots.	10
Results of syntheses	20a	For each synthesis, briefly summarise the characteristics and risk of bias among contributing studies.	10-11
	20b	Present results of all statistical syntheses conducted. If meta-analysis was done, present for each the summary estimate and its precision (e.g. confidence/credible interval) and measures of statistical heterogeneity. If comparing groups, describe the direction of the effect.	10
	20c	Present results of all investigations of possible causes of heterogeneity among study results.	10-11
	20d	Present results of all sensitivity analyses conducted to assess the robustness of the synthesized results.	11
Reporting biases	21	Present assessments of risk of bias due to missing results (arising from reporting biases) for each synthesis assessed.	11
Certainty of evidence	22	Present assessments of certainty (or confidence) in the body of evidence for each outcome assessed.	11 Table S 5-7
**DISCUSSION**			
Discussion	23a	Provide a general interpretation of the results in the context of other evidence.	11-13
	23b	Discuss any limitations of the evidence included in the review.	13-14
	23c	Discuss any limitations of the review processes used.	13-14
	23d	Discuss implications of the results for practice, policy, and future research.	14
**OTHER INFORMATION**			
Registration and protocol	24a	Provide registration information for the review, including register name and registration number, or state that the review was not registered.	7
	24b	Indicate where the review protocol can be accessed, or state that a protocol was not prepared.	7
	24c	Describe and explain any amendments to information provided at registration or in the protocol.	7
Support	25	Describe sources of financial or non-financial support for the review, and the role of the funders or sponsors in the review.	2-3
Competing interests	26	Declare any competing interests of review authors.	2
Availability of data, code and other materials	27	Report which of the following are publicly available and where they can be found: template data collection forms; data extracted from included studies; data used for all analyses; analytic code; any other materials used in the review.	7-8

From: Page MJ, McKenzie JE, Bossuyt PM, Boutron I, Hoffmann TC, Mulrow CD, et al. The PRISMA 2020 statement: an updated guideline for reporting systematic reviews. BMJ 2021;372:n71. doi: 10.1136/bmj.n71. For more information, visit: http://www.prisma-statement.or

Table S2. Risk of bias assessment – Analysis A: Risk of bias assessment on study level (a) and across studies (b).

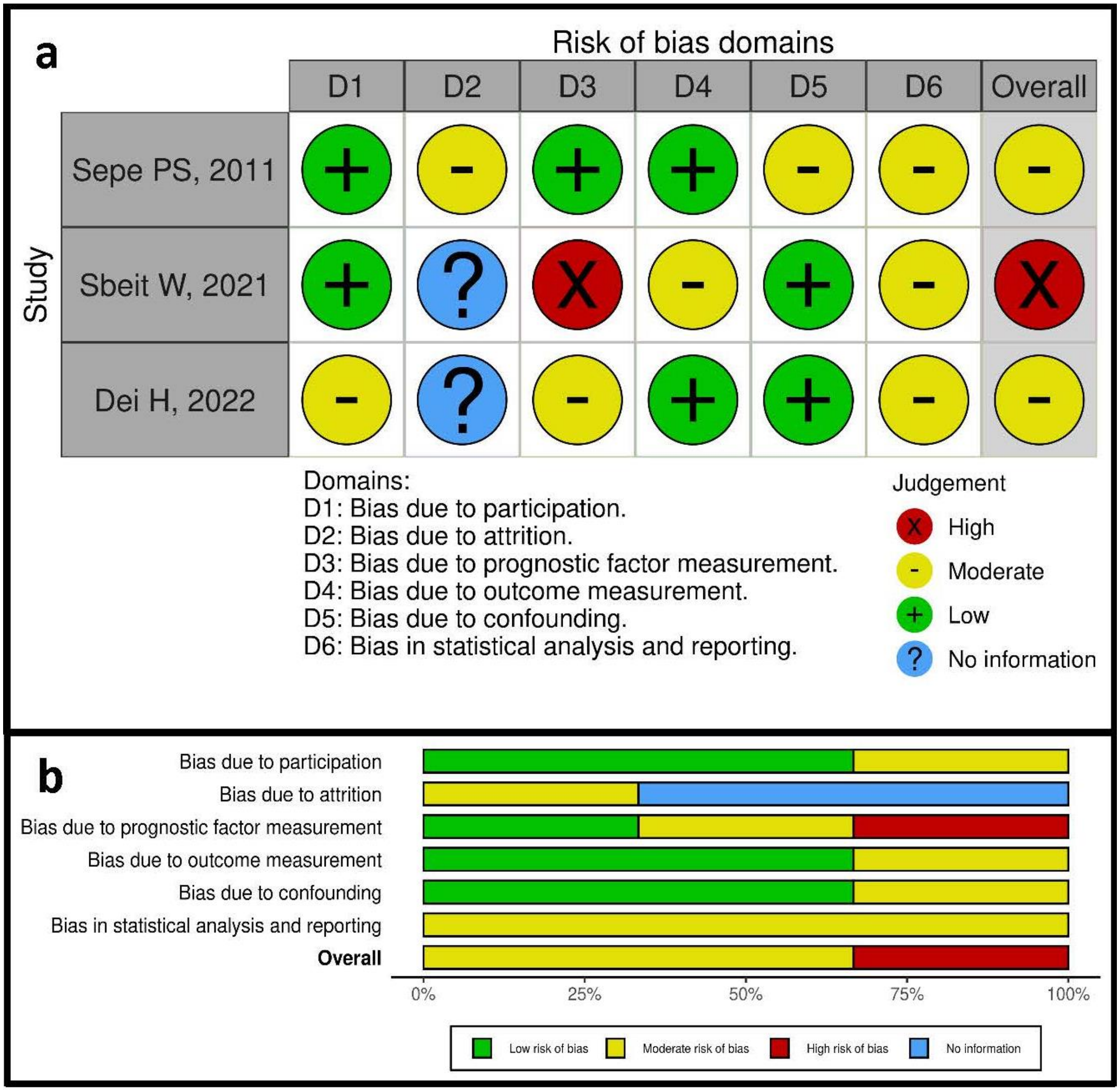

Table S3. Risk of bias assessment – Fatty pancreas among pancreatic cancer patients and controls: Risk of bias assessment on study level (a) and across studies (b).

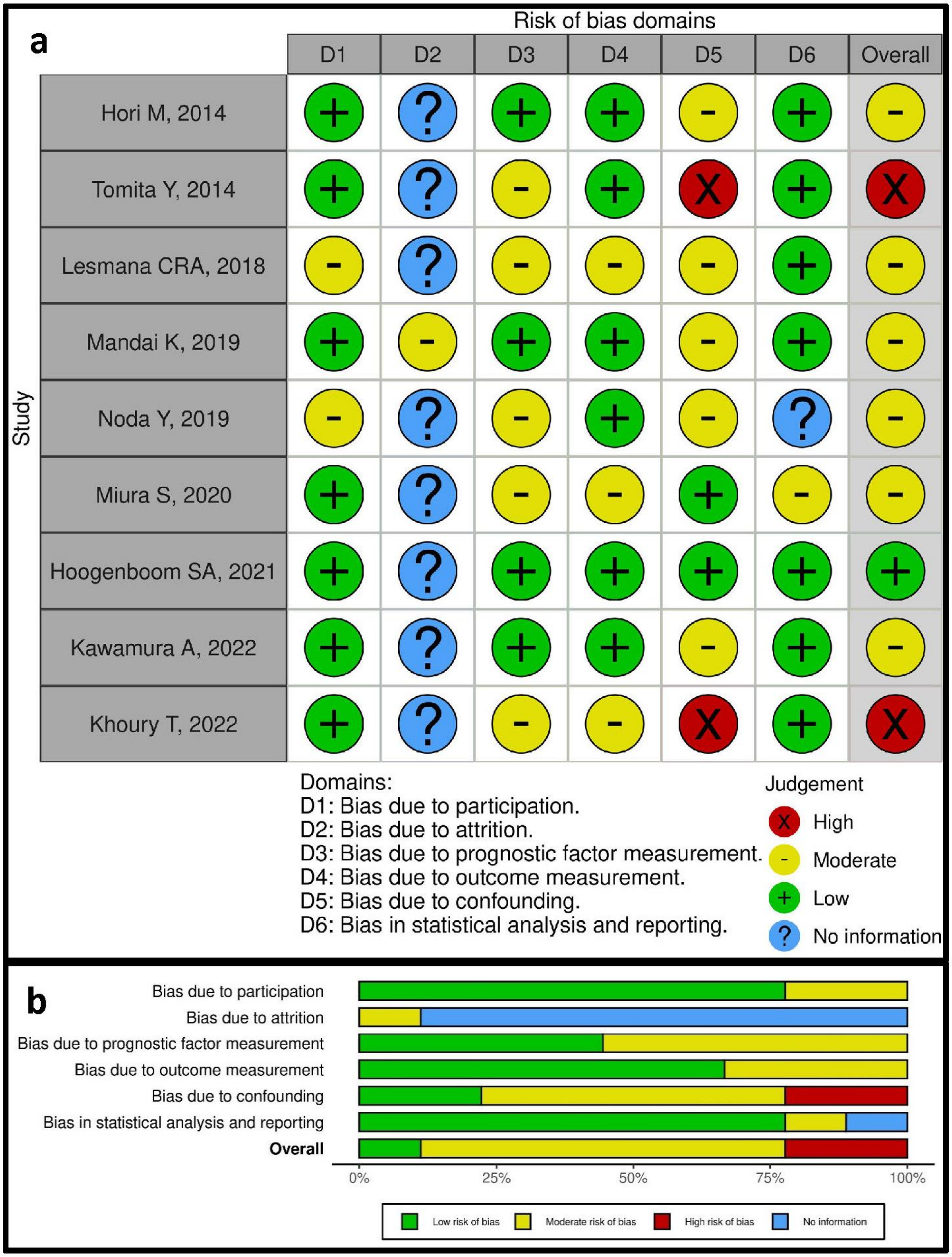

Table S4. Risk of bias assessment – Pancreatic cancer population: Risk of bias assessment on study level (a) and across studies (b).

0 No<br>1 Yes<br>3 Unclear<br>4 Not applicable	Was the sample frame appropriate to address the target population?	Were study participants sampled in an appropriate way?	Was the sample size adequate?	Were the study subjects and the setting described in detail?	Was the data analysis conducted with sufficient coverage of the identified sample?	Were valid methods used for the identification of the condition?	Was the condition measured in a standard, reliable way for all participants?	Was there appropriate statistical analysis?	Was the response rate adequate, and if not, was the low response rate managed appropriately?	Overall Risk of Bias
Mathur A. et al. (2011)	1	1	0	1	1	1	1	1	4	M
Gaujoux S. et al. (2012)	1	1	1	1	1	1	3	1	4	L
Hori M. et al. (2014)	1	1	1	1	1	0	1	1	4	L
Tomita Y. et al. (2014)	1	1	0	1	1	0	1	1	4	M
Kanno A. et al. (2018)	1	1	1	1	1	1	3	1	4	L
Lesmana C. et al. (2018)	1	1	0	3	1	3	1	1	4	M
Izumi Y. et al. (2019)	1	1	0	1	1	1	1	4	4	M
Mandai K. et al. (2019)	1	1	0	1	1	1	1	1	4	L
Noda Y. et al. (2019)	1	1	0	1	1	0	3	1	4	M
Miura S. et al. (2020)	1	0	0	1	1	1	3	1	4	M
Hoogenboom S. et al. (2021)	1	1	0	1	1	0	3	1	4	M
Kobashi Y. et al. (2021)	1	1	0	1	1	0	3	4	4	M
Kawamura A. et al. (2022)	1	1	0	1	1	0	1	1	4	M
Khoury T. et al. (2022)	1	1	0	1	1	1	1	1	4	L

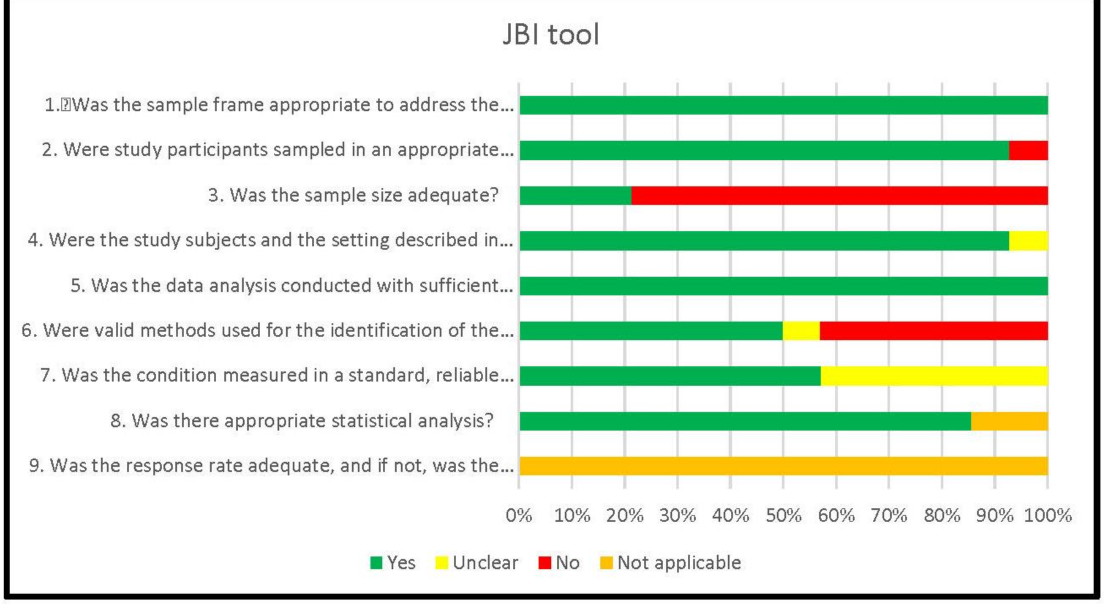

M = Medium risk; L = Low risk; JBI= Joanna Briggs Institute

Table S5. Evidence table of fatty pancreatic population and control group.

Certainty Assessment							№ of Patients		Effect		Certainty	Importance
№ of Studies	Study Design	Risk of Bias	Inconsistency	Indirectness	Imprecision	Other Considerations	[intervention]	[comparison]	Relative (95% CI)	Absolute (95% CI)		
3	Observational studies	Not serious	Not serious	Not serious	Serious[a]	None	42/234 (17.9%)	89/786 (11.3%)	**OR 1.32** (0.42 to 4.16)	**31 more per 1,000** (from 62 fewer to 234 more)	⊕◯◯◯ Very low	IMPORTANT

CI: Confidence interval; **OR:** Odds ratio

Explanations: a. Wide confidence interval

Table S6. Evidence table pancreatic cancer population and control group.

Certainty Assessment							№ of Patients		Effect		Certainty	Importance
№ of Studies	Study Design	Risk of Bias	Inconsistency	Indirectness	Imprecision	Other Considerations	[intervention]	[comparison]	Relative (95% CI)	Absolute (95% CI)		
9	Observational studies	Not serious	Serious[a]	Not serious	Very serious[b]	None	279/408 (68.4%)	275/1068 (25.7%)	**OR 6.13** (2.61 to 14.42)	**423 more per 1,000** (from 218 more to 576 more)	⊕◯◯◯ Very low	CRITICAL

CI: Confidence interval; **OR:** Odds ratio

Explanations: a. The heterogeneity was high; **b.** Wide confidence interval.

Table S7. Evidence table of pancreatic cancer population and control group.

№ of Studies	Certainty Assessment						Effect			Certainty	Importance
	Study Design	Risk of Bias	Inconsistency	Indirectness	Imprecision	Other Considerations	№ of Events	№ of Individuals	Rate (95% CI)		
14	Observational studies	Not serious	Serious[a]	Not serious	Not serious	None	441	868	Event rate 0.62 per 100 (0.42 to 0.79)	⊕⊕⊕◯ Moderate	CRITICAL

Explanations: a. The heterogeneity was high.

1. Schwarzer, G.; Chemaitelly, H.; Abu-Raddad, L.J.; Rucker, G. Seriously misleading results using inverse of Freeman-Tukey double arcsine transformation in meta-analysis of single proportions. *Res Synth Methods* **2019**, *10*, 476-483, doi:10.1002/jrsm.1348.
2. Stijnen, T.; Hamza, T.H.; Ozdemir, P. Random effects meta-analysis of event outcome in the framework of the generalized linear mixed model with applications in sparse data. *Stat Med* **2010**, *29*, 3046-3067, doi:10.1002/sim.4040.
3. Mantel, N.; Haenszel, W. Statistical Aspects of the Analysis of Data From Retrospective Studies of Disease. *JNCI: Journal of the National Cancer Institute* **1959**, *22*, 719-748, doi:10.1093/jnci/22.4.719.
4. Robins, J.; Greenland, S.; Breslow, N.E. A general estimator for the variance of the Mantel-Haenszel odds ratio. *Am J Epidemiol* **1986**, *124*, 719-723, doi:10.1093/oxfordjournals.aje.a114447.
5. Cooper HM, H.L., Valentine JC, editors. *The handbook of research synthesis and meta-analysis.*; Russell Sage Foundation: New York, 2009; Volume 2nd ed.
6. J. Sweeting M, J.S.A., C. Lambert P. What to add to nothing? Use and avoidance of continuity corrections in meta-analysis of sparse data. *Statistics in Medicine* **2004**, *23:1351–75.*, doi:10.1002/sim.1761.
7. Knapp, G.; Hartung, J. Improved tests for a random effects meta-regression with a single covariate. *Stat Med* **2003**, *22*, 2693-2710, doi:10.1002/sim.1482.
8. IntHout, J.; Ioannidis, J.P.; Borm, G.F. The Hartung-Knapp-Sidik-Jonkman method for random effects meta-analysis is straightforward and considerably outperforms the standard DerSimonian-Laird method. *BMC Med Res Methodol* **2014**, *14*, 25, doi:10.1186/1471-2288-14-25.
9. Jackson, D.; Law, M.; Rucker, G.; Schwarzer, G. The Hartung-Knapp modification for random-effects meta-analysis: A useful refinement but are there any residual concerns? *Stat Med* **2017**, *36*, 3923-3934, doi:10.1002/sim.7411.

10. Paule, R.C.; Mandel, J. Consensus Values and Weighting Factors. *J Res Natl Bur Stand (1977)* **1982**, *87*, 377-385, doi:10.6028/jres.087.022.
11. Veroniki, A.A.; Jackson, D.; Viechtbauer, W.; Bender, R.; Bowden, J.; Knapp, G.; Kuss, O.; Higgins, J.P.; Langan, D.; Salanti, G. Methods to estimate the between-study variance and its uncertainty in meta-analysis. *Res Synth Methods* **2016**, *7*, 55-79, doi:10.1002/jrsm.1164.
12. Agresti, A.; Coull, B.A. Approximate Is Better than "Exact" for Interval Estimation of Binomial Proportions. *The American Statistician* **1998**, *52*, 119-126, doi:10.2307/2685469.
